# Development and Evaluation of a New Security and Privacy Track in a Health Informatics Graduate Program: Multidisciplinary Collaboration in Education

**DOI:** 10.2196/mededu.9081

**Published:** 2018-12-21

**Authors:** Leming Zhou, Bambang Parmanto, James Joshi

**Affiliations:** 1 Department of Health Information Management University of Pittsburgh Pittsburgh, PA United States; 2 Department of Informatics and Networked Systems School of Computing and Information University of Pittsburgh Pittsburgh, PA United States

**Keywords:** information security, research projects, partnership and collaboration

## Abstract

**Background:**

The widespread application of technologies such as electronic health record systems, mobile health apps, and telemedicine platforms, has made it easy for health care providers to collect relevant data and deliver health care regimens. While efficacious, these new technologies also pose serious security and privacy challenges.

**Objective:**

The training program described here aims at preparing well-informed health information security and privacy professionals with enhanced course materials and various approaches.

**Methods:**

A new educational track has been built within a health informatics graduate program. Several existing graduate courses have been enhanced with new security and privacy modules. New labs and seminars have been created, and students are being encouraged to participate in research projects and obtain real-world experience from industry partners. Students in this track receive both theoretical education and hands-on practice. Evaluations have been performed on this new track by conducting multiple surveys on a sample of students.

**Results:**

We have succeeded in creating a new security track and developing a pertinent curriculum. The newly created security materials have been implemented in multiple courses. Our evaluation indicated that students (N=72) believed that receiving security and privacy training was important for health professionals, the provided security contents were interesting, and having the enhanced security and privacy training in this program was beneficial for their future career.

**Conclusions:**

The security and privacy education for health information professionals in this new security track has been significantly enhanced.

## Introduction

In most current health informatics, medical informatics, nursing informatics, and health information management (HI-MI-NI-HIM) education programs, there is either no dedicated security and privacy class or only one available course [1]. At the same time, however, it is widely accepted that security and privacy are crucial in the domain of health care. According to a study done by Staggers et al in 1999 [2], health care professionals recognized the importance of privacy and security and the need to enhance security and privacy education in this field.

Currently, the discussion on security and privacy issues continues in a variety of health care areas [3-6], especially in fields such as the electronic health record (EHR) systems, mobile health (mHealth) apps, and telemedicine platforms. These recent developments in health information technologies have created challenges in security and privacy that are far more serious than those seen just two decades ago.

According to the Office of the National Coordinator for Health Information Technology data brief released in 2016, 96% of hospitals in the United States possess certified EHR technology. Likewise, 84% of hospitals had adopted at least a basic EHR system with clinician notes in 2015, which is a 9-fold increase since 2008 [7,8]. In large hospitals, dedicated information technology (IT) teams protect patient health data, but in many smaller clinics, there are no dedicated IT personnel to manage EHR systems. This leaves a huge number of security vulnerabilities for attackers to explore.

The widespread adoption of new technologies such as mHealth apps and telemedicine systems makes security and privacy issues in health care more challenging and urgent since patients can easily share the confidential health data they receive from health care providers without knowing the security risks.

The cloud and health social networks, an emerging new frontier for health care delivery, pose new challenges in security and privacy as well. Currently, there are many social media tools available for patients and health care professionals, such as social networking platforms (Facebook, Twitter, LinkedIn), blogs, wikis, and photo and video sharing sites (Flickr, YouTube, Instagram). Patients use Twitter to obtain knowledge and exchange ideas and use Facebook to obtain social support, while health care professionals use LinkedIn and Twitter to communicate with colleagues and identify potential jobs [9]. The social media are great tools for communication and knowledge transfer. However, since patients have to share their personal information on social media when they seek information for their own disease, they should have serious concerns about their privacy [9]. When health care professionals share their own experience with colleagues via social media or upload patient records to the cloud, there is also risk of violating their patients’ information confidentiality [10].

Health care professionals are responsible for educating patients regarding the safe and ethical sharing of personal health records. Furthermore, health care professionals must properly handle all types of sensitive health records, such as personal genomic information, EHR data, and data collected with mobile apps or other various trackers and wearable sensors. In other words, the changing face of health IT and health care information management requires that we enhance the security and privacy education offered to health care professionals, especially to students in HI-MI-NI-HIM programs.

There are 3 typical approaches in enhancing security and privacy education in HI-MI-NI-HIM programs: (1) cross-listing existing security and privacy courses offered by computer science or information science departments, (2) adding a security and privacy course to the curriculum, or (3) addressing security and privacy issues in relevant courses. In the first approach, students in HI-MI-NI-HIM programs are required to have a solid background in computer science topics such as computer programming and computer networks. These prerequisites make the security and privacy courses inaccessible for many HI-MI-NI-HIM students. In the second approach, the major challenge for students is the connection between security and privacy theory and their application in health science. A single course can be used to introduce security and privacy theories but students do not have much chance to know the application of those theories in health care practice and health data management. The third approach is the opposite of the second approach. When instructors discuss specific health IT systems or health data management, they can describe the relevant security and privacy challenges; however, the instructors would not be able to go into the details of these challenges, the fundamental security and privacy theories behind those challenges, and the desired solutions.

In response to this current situation, a new information security and privacy track in a Health Information Systems (HIS) graduate program has been created in the Department of Health Information Management (HIM) in collaboration with the Department of Informatics and Networked Systems (INS) at the University of Pittsburgh (Pitt) with the goal of producing highly desired, well-trained security and privacy professionals in the domain of health care. Instead of simply modifying the curriculum by cross-listing a number of existing security and privacy courses or trying to cover every aspect of security and privacy in a single course, a number of significant curriculum changes have been made. For example, courses from INS were modified and introduced in this new track. Multiple existing courses have been enhanced with security and privacy contents. New labs, seminars, research projects, and internship activities have also been developed and offered to students. It is expected that this new track will enhance the security and privacy training to students in this graduate program.

## Methods

### New Track Creation

#### Modifying and Including Informatics and Networked Systems Graduate Courses

The courses in the HIS graduate program are classified by type as Health Informatics and Foundation, Health Management, or elective courses/thesis option. Of the 15 available Health Informatics and Foundation courses, students are required to take 10; of the 5 Health Management courses, they must take 2. For their remaining credits, students are allowed to take 2 additional electives or choose the thesis option. Students in the thesis option take 2 courses: Graduate Research Proposal and Graduate Research. Here, students obtain course credits by attending seminars and conducting research under the guide of a project mentor.

In developing the new security and privacy track, we introduced 2 required courses and 2 elective courses in the Health Informatics and Foundation category, offered in Pitt’s INS department. Required are Introduction to Security and Privacy (INFSCI 2150) and Security in E-Commerce (INFSCI 2731); Security Management and Computer Forensics (INFSCI 2621) and Developing Secure Systems (INFSCI 2620) are elective. These courses were chosen because they had rich contents in security and privacy, including both theoretical foundations (INFSCI 2150) and practical applications (INFSCI 2731, INFSCI 2621, and INFSCI 2620) in various fields. In addition, these 4 courses only required students to have general knowledge in computer systems and computer programming, which is also the prerequisite of our HIS graduate program. They did not expect students to have extensive knowledge in computer networks or cryptography. The instructors of these 4 courses have worked closely with faculty members in the HIS graduate program to create examples and case studies in these courses. The purpose was to contextualize these courses with current issues from the health care domain so that HIS students can gain an understanding of the most relevant security and privacy principles and technologies in the context of health care. For instance, INS instructors started to discuss Health Insurance Portability and Accountability Act (HIPAA) regulations and their impact to risk management, health data anonymization, health data security in the cloud, and secure health-related private data release. Table 1 shows the updated curriculum for the security and privacy track in the HIS graduate program at Pitt.

#### Enhancing Existing Courses

Some changes have been made to existing courses in the HIS graduate program. In Security, Privacy and Legal Issues in Health Systems (HRS 2421), we have added 2 guided discussion sessions, 2 student presentations, 2 scholarly papers, and 1 hands-on course project. In Telemedicine, Tele-rehabilitation, and e-Health (HRS 2432), security and privacy issues are described and discussed when each telemedicine platform is introduced in the class. Guest lecturers are invited to explain the details of certain systems, such as the security and privacy of patient records in web portals. In Topics in Health Care (HRS 2902), lectures regarding information security and privacy issues in health systems have been added. In Electronic Health Records (HRS 2490), a faculty member from INS delivers 2 lectures on the security and privacy of EHR records. Finally, 1 discussion session and 1 term paper on personal genomic information security have been added to Data Analytics and Its Applications in Genomics (HRS 2425).

#### Developing New Labs

Six new educational lab modules have been introduced into various classes throughout the new track. These lab modules include lifecycle management in cloud and health social networks, security policy and auditing issues in the health care environment, authentication and identity management, access control for EHR systems, secure mobile apps and social networks in health care, and HIPAA compliance management. All these labs are closely related to pressing security and privacy issues in the domain of health care such as the cloud, social media, mobile app, identity management, access control, and HIPAA compliance. Instructors from the INS and HIM departments met and discussed the content of these lab modules. Each instructor took the lead of 1 lab module according to his/her expertise. For instance, Valerie Watzlaf in the HIM department guided students to investigate the HIPAA compliance of existing telemedicine systems; LZ led students to evaluate the security of a new EHR system created for the FOCUS Pittsburgh Free Health Clinic (FPFHC) and BP trained students to investigate the security of mobile apps.

For students in HI-MI-NI-HIM programs, EHR is one system they should be familiar with. Lab exercises were created for them to get familiar with multiple EHR systems. In the Electronic Health Records (HRS 2490) class, the Virtual Lab system developed by the American Health Information Management Association was used to teach students how to use EHR systems such as the one made by Cerner to manage and protect patient data. We also introduce the OpenEMR (open-emr.org) to students, which is freely available to everyone. Students can make changes on the OpenEMR system to see the differences in the output, which is quite beneficial for them to identify the impact of different authentication methods.

#### Developing New Seminars

Four new seminars have been created. These new seminars are typically related to specific research projects led by faculty members in the HIM and INS departments, with topics in high assurance electronic health (eHealth) and health IT infrastructure, security and privacy in the cloud and health social networks, secure health care cyber-physical systems, and advanced topics in secure health care information systems. In these seminars, both instructors and students read the current literature and gave presentations that are followed by extensive discussion in the class.

#### Designing Small-Scale Research Projects

Small-scale research projects are created by faculty members and provided to students. Some examples include the security features of a new health IT system created for a free clinic, the security of published mobile health apps, and projects in topics such as access control, social network, and cloud security.

LZ has been working closely with FPFHC to create a health IT system for the clinic. Everything is created from scratch, which provides abundant security and privacy research opportunities for students. Students involved in this project can test different security measures and privacy policies and manipulate fake user accounts and medical records to determine the security of the implemented system. They have the opportunity to go through the whole life cycle of secure system development and testing.

JJ did extensive research in security and privacy such as role-based access control (RBAC), temporal access control, geo-social-RBAC, anonymization, identity threats, and security and privacy issues in social network systems and the cloud computing environment [11-22]. In this new security and privacy track, JJ has created research projects and test beds for graduate students to conduct research projects in access control, social network, and cloud security.

BP has led the development of multiple innovative mHealth systems and telemedicine platforms, including the iMHere system for mHealth and VISYTER (Versatile and Integrated System for Telerehabilitation) for telemedicine [23,24]. In this track, he has created research projects for graduate students to investigate the security issues in existing telehealth systems and mobile health apps.

**Table 1:** Curriculum of the Health Information Systems graduate program, Security and Privacy Track.

Course type and number		Course title	Credit
**Prerequisites**			
	—^a^	Computer programming	3
	—	Statistics	3
**Health Informatics and Foundation courses (select 29-30 credits)**			
	HRS 2420	Introduction to Health Information Systems	3
	HRS 2421	Security, Privacy, and Legal Issues in Health Systems	3
	HRS 2422	Computer Programming for Health Informatics	3
	HRS 2424	Data Base Management in Health Care	3
	HRS 2428	Software Engineering Project Management	3
	HRS 2439	Health Information Systems Internship	3
	HRS 2490	Electronic Health Records	3
	HRS 2423	Cloud Computing, HL7^b^, and Analytics in Health Care	3
	HRS 2425	Data Analytics and Its Applications in Genomics	3
	HRS 2426	Evaluation of Classification Systems	3
	HRS 2431	Evaluation Methods in Health Information Systems	3
	HRS 2432	Telemedicine, Tele-Rehabilitation, and e-Health	3
	HRS 2434	Business Issues/Data Analytics in Health Care	2
	HRS 2901	Introduction to Research Methodology	3
	HRS 2910	Statistical Applications in Health and Rehabilitation	3
	INFSCI 2150	Introduction to Security and Privacy	3
	INFSCI 2731	Security in E-Commerce	3
	INFSCI 2621	Security Management and Computer Forensics	3
	INFSCI 2620	Developing Secure Systems	3
**Health Management courses (select 6 credits)**			
	HRS 2435	Financial Management Foundations	3
	HRS 2445	Human Resource Management	3
	HRS 2454	Lean Six Sigma for Healthcare Management	3
	HRS 2902	Topics in Health Care	3
	HRS 2905	Ethical Issues in Health Care	3
**Thesis option (6 credits)**			
	HRS 2924	Graduate Research Proposal	2
	HRS 2925	Graduate Research	4

^a^Not applicable.

^b^HL7: Health Level 7.

#### Providing Internship Experiences

We always encourage students to seek internship experiences in different health care organizations. For instance, many graduate students have the opportunity to work with organizations such as the University of Pittsburgh Medical Center (UPMC) hospitals and Veterans Affairs Pittsburgh Health System as interns. Currently, the HIM department at Pitt has more than 80 active industry partners including multiple UPMC hospitals and various nonprofit health organizations. Students in this new track are specifically encouraged to have security- and privacy-related internship experience with these industry partners.

### Evaluation of the New Track

Since the course materials have just recently been implemented, we can offer only a preliminary evaluation report. Pitt’s Collaborative for Evaluation and Assessment Capacity worked closely with the project team to perform the evaluation, which included collecting data according to the project objectives in each course/activity as well as evaluating the impact of project activities on learning outcomes. All new items in the track will be evaluated through observation, pre/post comparisons, surveys, and/or feedback from students and faculty.

In general, evaluation activities involve the following:

- Collecting information regarding student satisfaction and perception toward the new course materials, training approach, and other activities through surveys
- Measuring student learning results through performance in courses, projects, or internships and feedback from course instructors or internship supervisors
- Identifying any evidence that students are applying acquired skills in subsequent courses using behavior change checklists
- Documenting any evidence that students are using new knowledge and skills beyond coursework by conducting postgradation/employment surveys

## Results

In Fall 2015, 2 courses, Topics in Health Care (HRS 2902) and Telemedicine, Tele-rehabilitation, and e-Health (HRS 2432), were enhanced with security and privacy content. Two brief and informal questionnaires designed by the course instructors were distributed to students in the class at the end of the semester. The questionnaires were aimed at obtaining a general measure of students’ opinions on the new security and privacy contents and pedagogy. In HRS 2902, 3 brief questions were asked, and 4 students responded (see Textbox 1). In the HRS 2432 class, 4 questions were asked and 13 students responded (see Textbox 2).

During Spring 2016, a Web-based survey was implemented in all security- and privacy-enhanced courses in the new track. This survey asks questions regarding students’ opinions on security and privacy in health systems before and after taking one security- and privacy-enhanced course. By December 2017, 65 students had participated in the survey, and 55 of them provided their answers beyond the basic demographic information. The results reported below are based on the analysis of these 55 students’ answers. Each of these 55 students had taken at least one of the security- and privacy-enhanced courses, such as HRS 2421, HRS 2432, HRS 2902, or HRS 2425. Among these student, 56% (31/55) of students were from the HIS master’s program, 16% (9/55) were from the Health Care Supervision and Management master’s program (HSM), 4% (2/55) students were in the Rehabilitation Science PhD program, and 22% (12/55) were from other master’s programs such as Prosthetics and Orthotics (PO, 10/55, 18%), Rehabilitation Science and Technology (RST, 1/55, 2%), and Nutrition and Dietetics (ND, 1/55, 2%). Details about these graduate programs can be found at www.shrs.pitt.edu/programs. Please note that students in programs other than the HIS master’s program are not required to take security- and privacy-related courses and their future work typically does not require them to have security and privacy knowledge either.

Before performing the analysis on the collected data, we noticed that the answers from 3 students were not consistent. Since this Web-based survey was only conducted at the end of the course, instead of before and after the course, some students were confused by the change of terms in the questions for indicating time. Terms used in the questions or statements included now versus then, before versus after, nothing versus thinking back, and after versus prior to. They put some answers to precourse into the box for postcourse and the other way around in questions. For instance, in the now/then questions, these 3 students indicated that they knew significantly more about information security now (after they took the course) and they wanted to take more security courses in the future. One of them actually had already taken more than 1 security- and privacy-related courses in the new track. However, their answers to the before/after questions showed the opposite (did not learn much from the course and did not want to take more security- and privacy-related courses). In this case, we corrected the position of these 3 students’ answers to make them internally consistent, assuming their first answer to this type of question is correct.

For the first 2 questions (Q1 and Q2) in the survey, we simply reported the percentage of options chosen by these 55 students since they were basic fact checking after they took the course(s). The mean and standard deviation (SD) were calculated for survey items with 5 options (either from 1=not very much to 5=very much or from 1=strongly disagree to 5=strongly agree). The distribution of the answers from these 55 students were checked and they were not normally distributed (*P*<.05 in the Shapiro-Wilk test on each item). Therefore, the answers before and after taking the course are compared with a nonparametric Wilcoxon signed-rank test on related samples. The results are summarized in Textbox 3 and Table 2.

**HRS 2902 questionnaire and answers.**

*Please rate your concern in security and privacy issues in the health systems before you attended the security and privacy lectures (1=not concerned at all, 10=extremely concerned)?*

Students responded with ratings of 6, 7, 8, and 9. This indicates that these students had different levels of concern in security and privacy issues in the health systems before attending the security and privacy lectures.

*Did the security and privacy lectures provide you with ideas and examples for security and privacy assurance in health care?*

All 4 students answered yes.

*Do you plan to take further courses or have the desire to learn more on security and privacy in the future?*

All 4 students answered yes. Some even indicated the specific topics they would like to learn more about such as access control, authentication, and encryption.

**HRS 2432 questionnaire and answers.**

*What is your perspective on privacy and security in telehealth before taking this course?*

Seven students (54%) believed security and privacy were important, one student (8%) had concerns, and 5 students (39%) did not see privacy and security as a serious issue.

*What is your view of privacy and security in telehealth after taking this course?*

Twelve students (92%) expressed that security and privacy are very important. One student (8%) did not believe security and privacy to be significant issues.

*Please describe how beneficial the following approaches are in increasing your knowledge of privacy and security in telehealth:*

*Security and privacy issues are mentioned throughout the course and connected to specific projects in telehealth.*

Twelve students (92%) believed that this approach was beneficial; one student did not answer.

*Security and privacy issues are presented as a specific topic/module by guest lecturers.*

Twelve students (92%) believed that this approach was very helpful since these guest lectures provided real world cases demonstrated how security and privacy concepts were applied in current systems. One student (8%) believed this approach was fair.

*Security and privacy issues are considered as a part of the final project.*

Twelve students (92%) believed that this approach was beneficial. One student (8%) believed that this approach was not beneficial.

*Would you be interested in taking a more advanced course in privacy and security?*

Ten students (77%) answered yes, 2 students (15%) answered no, and one student (8%) was unsure.

**Questions 1 and 2 and students’ answers.**

*Q1. I am considering/would consider entering the health privacy and security track within my degree program*.

Fifteen students (27%) chose yes, and 17 students (31%) chose no. Three student (6%) answered that they were already enrolled, and 19 students (35%) answered maybe.

*Q2. Did you take any other courses this academic year that included security and privacy modules?*

Twenty-two (40%) students answered yes, and 33 students (60%) answered no. The courses these students took were HRS 2425, HRS 2432, HRS 2903, and HRS 2421.

**Table 2:** A summary of the answers to Question 3 and responses to 6 statements on the Web-based survey.

Questions/statements		Before, mean (SD)		After, mean (SD)		*P* value
Q3. How much did/do you know about security and privacy in health care before/after taking the course? (1=not very much, 2=a little, 3=some, 4=a lot, 5=very much) (n=51)		2.29 (1.026)		4.87 (1.833)		<.001
**Before/after taking this course with security and privacy content, rate the following statements (1=strongly disagree, 3=neither agree nor disagree, 5=strongly agree)**						
	S1. Security and privacy content is interesting (n=41)		3.29 (0.891)		3.77 (0.890)	.01
	S2. Receiving training in security and privacy is worth the effort (n=42)		3.64 (0.821)		4.15 (0.659)	.006
	S3. Improving knowledge of security and privacy is needed to ensure cybersecurity in today’s health fields (n=42)		3.81 (0.862)		4.39 (0.614)	<.001
	S4. Security and privacy training is a beneficial addition to my coursework (n=41)		3.76 (0.830)		4.13 (0.647)	.02
	S5. I am planning to enter a career that will require knowledge of security and privacy of health information (n=41)		3.51 (1.028)		3.96 (0.779)	.003
	S6. I would like to take more courses with security and privacy content (n=41)		3.20 (0.954)		3.68 (0.911)	.002

The answers to the question (Q3) before and after taking the course are statistically different (*P*<.001), suggesting that students know significantly more about security and privacy after taking one or more security-enhanced courses in this new track. Their opinion of security-enhanced courses also shifted in a positive direction after they took those courses, and all changes were statistically significant according to the related-samples Wilcoxon signed-rank test. In other words, after they took the courses, the students had a statistically significant stronger agreement with the 6 statements (S1 through S6), which indicated that this education program is effective in terms of improving students’ knowledge in security, desire to learn more in this field, and interest in working in this area.

An independent-samples Kruskal-Wallis test was performed to determine the opinion difference between students who had taken multiple security-enhanced courses versus one such course after they took the course. The result indicated that students who had taken multiple security-enhanced courses expressed significantly higher agreement on 2 statements: S1—Security and privacy content is interesting (*P*=.01) and S3—Improving knowledge of security and privacy is needed to ensure cybersecurity in today’s health fields (*P*<.001), which was consistent with their behavior in that they took multiple security-enhanced courses in the track.

A one-way analysis of variance (ANOVA) was performed to determine the opinion difference among students who were in different programs: HIS (HIS master’s program and Rehabilitation Science PhD program, n=33), HSM (n=9), and others (RST, ND, and PO, n=12). The ANOVA result indicated that students from different academic programs had statistically significant difference in their answers to 2 statements: S1 (Security and privacy content is interesting, *F*=5.192, *P*=.01), and S6 (I would like to take more courses with security and privacy content, *F*=4.113, *P*=.02). More specifically, students from the HIS program expressed significantly stronger agreement on S1 than students from HSM, PO, RST, and ND both before (*P*=.008) and after (*P*=.04) they took the security-enhanced courses; students from HIS also indicated significantly stronger agreement on S6 than students from HSM, PO, RST, and ND programs after they took the courses (*P*=.03). In other words, students in the HIS program considered security-related topics interesting before and after the courses and they had significantly stronger desire to take more courses with security and privacy content.

## Discussion

### Principal Findings

In this project, we used various approaches to enhance security and privacy materials in a new track and deliver the new materials to graduate students in the health science programs without placing a significantly heavier burden on students. More specifically, instead of simply adding one or more courses into an existing curriculum, we modified existing security and privacy courses; added security and privacy contents into other relevant courses; developed new labs, seminars, and research projects in the field of security and privacy; and provided internship experiences.

To evaluate the outcome of our approach, we used multiple surveys and collected data from 72 students who took our security- and privacy-enhanced courses. Although the backgrounds of these students were different, including their knowledge in security and privacy before taking these courses, the evaluation results indicated that students learned a lot in these courses, considered security and privacy content interesting and worth the efforts, and had the desire to learn more. We also noticed that students in programs other than HIS had a relatively lower desire to take more security and privacy courses. In other words, even though it is well accepted that security and privacy are critically important for health science students, not all students are willing to receive extensive training on this topic. Therefore, this enhanced security and privacy training cannot be required for all students in this field.

One essential component in creating the graduate security and privacy track described in this article was close collaboration between the HIM and INS departments at Pitt. This education collaboration started in 2009 in a National Science Foundation education project aimed at improving students’ computational thinking ability. In that project, we had monthly meetings where all project team members sat to discuss progress. From that project, HIM and INS have gone on to develop a stronger collaborative relationship with respect to both student education and research.

The existence of this close collaboration between faculty members in HIM and INS has made it possible to make changes in course materials and schedules according to the requirements of the other department. For instance, HIM could request that the instructors of the INS courses include examples from the health care domain in their lectures or labs so that the materials are more accessible for students in the HIS graduate program.

Furthermore, Pitt’s many leading security and privacy researchers have helped augment this project’s success. These researchers have been recruited to help develop new labs, seminars, and research projects that ultimately serve as an advantage to this new track.

The HIM department at Pitt has active collaborations with a large number of industry partners. Thus, it is convenient for students to seek internship experiences in these organizations since there are many positions available close to Pitt’s campus. This is also an important factor in the creation of this new security and privacy track.

### Comparisons With Previous Work

Although researchers, educators, and health care practitioners recognized the importance of security and privacy education in health science and medical training a long time ago, many current HI-MI-NI-HIM programs still do not provide sufficient training in health information security and privacy. Some programs do not have any security- and privacy-related courses or only have a course on HIPAA regulation and other legal issues, a course about information governance, a course specifically about health information security and privacy, or a few cross-listed courses offered by a computer science or information science department. There are also some health information security and privacy certificate programs which offer one or multiple security and privacy courses.

One reason for this current situation is that it is challenging to add new content to the HI-MI-NI-HIM programs because their curriculum is full with many other essential courses on data analytics, health IT systems, statistics, databases, computer programming, health care systems, data management and regulation, quality management, coding, leadership, clinical education, finance, and internship. Therefore, if the new materials cannot be integrated into the existing courses, labs, and internships, students would not have time to learn them.

Our project offers a unique approach. The security and privacy materials are organized and distributed into multiple courses, labs, seminars, small-scale research projects, and internship. Students learn the security and privacy knowledge in specific health care contexts and can directly apply the knowledge to their professional practice.

### Limitations

The evaluation was only on a portion of the entire project. Evaluations of other parts are currently ongoing. As we mentioned earlier, we are also using other evaluation approaches such as observation, focus group, and feedback from students and faculty members to determine the effectiveness of this new track. Therefore, the reported results are still considered as preliminary and the sample size is not very large. The total number of study participants was 72. Even so, these results are consistent and valuable for making adjustments in the implementation of this new security and privacy track in our future course offering.

We noticed that a few students were confused by the terms now and then and before and after in the survey questions and statements. We should have used the terms consistently. To avoid this problem, a better solution would be to ask students to complete the same survey before and after they took the course.

### Future Work

In the collaboration with the INS department at Pitt, we are exploring another approach for enhancing security and privacy education in health care: providing health science training to graduate students in the INS department with a security and privacy concentration. We will perform evaluation and comparison to determine the effectiveness of this approach compared to the approach described in this article.

A website (www.sis.pitt.edu/sahi/index.html) was created to provide further details about this security and privacy track to people who are interested in creating a similar track in their programs. The website also provides information about teaching health science to information science graduate students.

### Conclusions

In close collaboration with the INS security and privacy faculty, we have created a new security and privacy track in the HIS graduate program at Pitt. Enhanced courses, course modules, labs, seminars, and research projects are currently offered to graduate students in this program. Evaluation results were generated from surveys completed by 72 students, and they can be used to guide the further implementation of this new track. We believe this program will generate health informatics professionals with stronger security and privacy skills who will be ready to contribute to the protection of critical health data.

## Abbreviations

ANOVA: analysis of variance
eHealth: electronic health
EHR: electronic health record
FPFHC: FOCUS Pittsburgh Free Health Clinic
HI-MI-NI-HIM: health informatics, medical informatics, nursing informatics, and health information management
HIM: Health Information Management
HIPAA: Health Insurance Portability and Accountability Act
HIS: Health Information Systems
HRS: Health and Rehabilitation Sciences
HSM: Health Care Supervision and Management
INS: Informatics and Networked Systems
IT: information technology
mHealth: mobile health
ND: Nutrition and Dietetics
Pitt: University of Pittsburgh
PO: Prosthetics and Orthotics
RBAC: role-based access control
RST: Rehabilitation Science and Technology
UPMC: University of Pittsburgh Medical Center
VISYTER: Versatile and Integrated System for Telerehabilitation
